# Electrochemotherapy (ECT) and irreversible electroporation (IRE) -advanced techniques for treating deep-seated tumors based on electroporation

**DOI:** 10.1186/1475-925X-14-S3-I1

**Published:** 2015-08-27

**Authors:** Damijan Miklavcic, Rafael V Davalos

**Affiliations:** 1 University of Ljubljana, Faculty of Electrical Engineering, Ljubljana, 1000, Slovenia; 2 School of Biomedical Engineering and Sciences, Virginia Tech - Wake Forest University, Blacksburg, VA 24061, USA

## 

When a cell is exposed to a sufficiently intense electric field (for a sufficient duration), local defects in the cell membrane appear and become permeable to molecules that otherwise cannot pass through it. This phenomenon is commonly known as electroporation but additional terms electropermeabilization and PEF (i.e. pulsed electric field) treatment are commonly used as well (the latter mainly in food processing area). In reversible electroporation, cell membrane permeabilization to molecules otherwise deprived of transmembrane transport mechanisms is a transient phenomenon as after some time membrane reseals and reestablishes its homeostatic semipermeable properties, allowing ions and other small molecules to passively cross. The cell can thus survive, though if the molecular transport disruptions to homeostasis is too great, the cell will die and the process is termed irreversible electroporation. During this period of increased membrane permeability, if the cell is surrounded by chemotherapeutics like bleomycin and cisplatin they are able to enter the cell and access their intracellular targets. In this way, the increased transport of chemotherapeutics due to electroporation increases the cytotoxic effects of certain drugs through potentiated mass transport permitted by the state of increased membrane permeabilization. This process is commonly referred to as electrochemotherapy and involves the combination of reversible electroporation and chemotherapy, allowing the latter to be successfully used as local tumor treatment [1].

If the number of pulses is too great, their duration too long, or the amplitude of the field to which the cell is exposed too intense, the cell dies due to irreversible electroporation presumably due to a loss of homeostasis. Irreversible electroporation was introduced as an ablation method for normal and tumor tissue and quickly gained much attention due to its non-thermal nature, enabling operators to spare critical structures of the tissue matrix, such as vessels, while successfully eradicating tumor cells. The term "non-thermal" refers to the mechanism of IRE, since cell death is due to membrane electroporation and not a temperature increase of the tissue; although local temperature increases can occur around the electrodes especially if many pulses are administered.

In this editorial, we highlight recent efforts to address some of the current challenges associated with treating deep-seated tumors using electroporation-based therapies, how several of the papers in this special issue address those challenges, and point readers to several excellent review articles in the field for further background information [2-6]. This special issue is a result of a special session, which was organized by COST TD1104 Action EP4Bio2Med (http://www.electroporation.net) at the MBEC2014 6^th ^European Conference of the International Federation for Medical and Biological Engineering held in Dubrovnik, Croatia from September 7-11, 2014. It hosted a number of distinguished speakers from around the world. The presentations were grouped into three topics: *clinical experience *using electroporation based treatment of deep-seated tumors, *modeling and treatment planning *for deep-seated tumors using electroporation, and *prognostic imaging *in deep-seated tumors using electroporation.

Current challenges that have been identified during the special session when discussing either IRE or ECT of deep-seated tumors are: tissue conductivity determination (necrotic regions, vasculature, micro-heterogeneities); conductivity changes due to electroporation (amount and dynamics); threshold determination for electroporation (reversible, irreversible) also as a function of pulse parameters (duration and number of pulses); and the accuracy needed for treatment planning, positioning of electrodes, which consequently dictates also accuracy and robustness of a treatment plan. Many of these challenges have been further addressed in papers presented in this special issue and although they do not offer immediate and ultimate responses to all of the challenges identified, they do show possible directions on how to approach many of them. However, it is becoming clear that we will only be able to make this technology platform available for widespread use by clinicians and provide the best outcome and benefit for the patient through a holistic analysis of: imaging, treatment planning, and clinical feedback.

From the point of view of the clinician, the challenge is that currently it takes too long when compared to other minimally-invasive procedures such as microwave or RFA due to the requirement of multiple needles for IRE whereas in RFA and microwave, only one probe is inserted.

Electroporation based therapies currently require accurate placement of two of more electrodes within the region of interest. At the present time IRE procedures tend to extend for significantly longer time compared to other ablation technologies. Despite the use of ultrasound (US) during the electrode placement process, it is challenging and time-consuming; this is mainly due to the inherent nature of tumors treated with IRE which are surrounded by critical structures. More extensive use and availability of treatment planning will aide physicians with the electrode placement process given that it illustrates, on a physics-based model, what the most optimal and feasible electrode placement procedure may be.

The first paper by Liberti and colleagues describes modeling the positioning of single needle electrodes for the treatment of breast cancer in a clinical case [7]. The paper elegantly correlates numerical simulations with an actual clinical trial outcome--a topic that remains presently underdeveloped in the literature--considering the great variability present between cases and requisite development of a good cohort of trials to improve treatment planning for tumor tissue in clinical settings. It uses the resistance data and histology outcomes from a breast tumor that underwent resection after performing ECT in order to determine the theoretical oncologic outcome that would have been encountered from the ECT. Their study analyzed the feasibility of using ECT to treat patients with histologically proven unifocal ductal breast cancer. Due to the tumor being located in a relatively low-conductivity fat tissue, their numerical model reveals reduced electric field intensity induced inside the tumor with certain electrode placement geometries. Their study agreed with animal results by Neal and others using experiments in the mammary fat pad of mice and single insertion device that the field distribution is heavily dependent on the surrounding environment [8,9]. The results presented by Liberti et al. show that the effectiveness of the treatment can be enhanced through the careful placement of the electrodes and that treatment models could be used to identify better electrode configurations in planning the experimental protocols.

In treating deep seated tumors either during open surgery or percutaneously in liver or other organs due to high voltage (up to 3000 V) and consequently high currents (up to 50 A) delivered pulses could potentially interfere with cardiac activity [10]. Synchronization of delivery of electroporation pulses with ECG proved to reduce risk of interference. Mali et al. only found clinically irrelevant changes in heart rate variability (HRV) parameters after ECT procedure: a decrease in median values of the mean NN interval, a decrease in the low-frequency and in the normalized low-frequency component, and an increase in the normalized high-frequency component [11].

In addition to simplifying procedures by using a single insertion device, as described in the previous paper, treatment planning can be used to mitigate some of the challenges with delivering effective ECT, IRE, and EGT therapies. Several studies have been conducted to create more accurate treatment planning models [12-15] and pre-clinical models have demonstrated their effectiveness [16,17].

The pioneering work by Groselj, G Sersa and colleagues demonstrated the power of coupling treatment planning with a navigation system for the treatment of tumors using electroporation-based therapies [18]. By integrating a treatment plan with a navigation system, the navigation system was able to help the surgeon to identify the tumors and accurately position five long needle electrodes for treatment of a patient with head and neck tumors.

Although treatment planning is a powerful tool to aid clinicians in optimizing therapy, there are tremendous challenges with predicting the treatment outcome. For a given set of pulse parameters and tissue type, treatment outcome is determined by the electric field distribution [19]. However, the impedance distribution is difficult to predict *a priori *due to local heterogeneities present within the tumor tissue (e.g., necrotic core), tissue-to-tissue variability [20], patient-to-patient variability, and impedance changes of the local tissue due to electroporation itself [21,22]. Electroporation-induced dynamic tissue conductivity plays a significant role in redistribution of electrical conductivity and thus electric field and ablation zone [23,24]. When cells become electroporated, their capacity to behave as dielectrics is dramatically reduced, thus facilitating improved electric current flow through cellular regions relative to non-electroporated tissues [25].

Numerical methods are most commonly used for pre-treatment planning of electroporation-based therapies because the complex geometries of the tumor and surrounding structures disallow the development of analytical estimation techniques. In order to accurately model the electric field distribution, the impedance changes due to electroporation and tissue heterogeneities need to be characterized prior to treatment. Accordingly, studies employing numerical models for pre-treatment planning have accounted for the dynamically changing electrical properties of tissue [26-31], as well as the heterogeneous impedance in multiple geometries in the local tumor environment [20]. However, the presence of local heterogeneities in tumors (such as necrotic core), tissue-to-tissue variability and patient-to-patient variability further complicates the prediction of the impedance distributions.

Relevant inherent tissue heterogeneities include highly localized effects such as those due to the presence of diverse interstitial constituents including macro- and microvasculature, connective tissue, general cellularity, cellular morphology, and discrete functional organ units such as proximal convoluted tubules or glomerular capsules. Furthermore, certain tissues may contain stochastic or organized aspects that will have varying extent of an effect on electric field distributions, including presence of randomly distributed calcifications, as well as structural tissue orientations that exhibit anisotropic effects to electric field distribution. While this will occur for several tissue varieties, the strongest effect is clearly exhibited in muscle tissue where the high conductivity along muscle fibers compared to perpendicular to muscle fibers imparts a strong effect on electric field distribution and ablation shape [32]. Furthermore, it is too difficult to consider all of the subcellular to cellular-level variability within a targeted environment, and thus effective bulk tissue conductivity is used as a metric to approximate the overall conductivity for a particular tissue type. The electrical conductivity for different tissue types may more readily be identified and incorporated separately on predictive numerical models.

More recently, theoretical studies on high-frequency bipolar pulses (≈ 1 us) have shown that the use of bursts of such pulses helps achieve macroscopically homogeneous distributions on heterogeneous geometries [33,34].

The paper by Bhonsle et al. extends the advantages of high-frequency pulses and shows that they mitigate the dynamic impedance changes during electroporation therapy while achieving similar sized lesions as conventional unipolar pulses through the use of higher voltage pulses [35]. This paves way for modeling the electric field distribution in tissue using the analytical solution of the Laplace's equation that is valid for a homogeneous medium of constant conductivity. High-frequency pulses could have implications of decreased tissue-to-tissue variability during electroporation therapy, with one mathematical general solution fitting all tissues.

Although treatment planning is powerful, the tools are not available for all clinicians using electroporation-based therapies. The paper by Marcan and colleagues demonstrates how a web-based tool could allow users to build a 3D model of the target tissue from uploaded medical images and then simulate the electric field distribution for treatment planning [36]. This could be an invaluable tool for clinicians to carry out patient-specific treatment planning for electroporation-based treatments without advanced technical knowledge of the field.

Considering the challenges with predicting the impedance distribution beforehand for treatment planning, researchers are using imaging techniques to assess the impedance distribution within the tissue. Researchers have used medical images (e.g. MRI) in the past to determine precise anatomical geometries of the tumor and surrounding tissues and assign tissues with appropriate electrical properties [26,16,17,37]. However, as mentioned before, knowledge of these properties prior to treatment is difficult to ascertain.

The electric field distribution generated in a given tissue determines the outcome of electroporation based therapies. Previously, *in situ *imaging methods, such as electrical impedance tomography (EIT) [31] and magnetic resonance electrical impedance tomography (MREIT) [38], have been proposed to monitor the electric field distribution in real-time. Despite promising results, these methods are not yet well adopted for electroporation treatments due to the complexities involved in their implementation [39].

The paper by Igor Sersa and colleagues demonstrates a novel approach of using current density imaging (CDI) to monitor electroporation-based therapy [40]. Their approach overcomes several of the technical challenges associated with using other imaging modalities. They successfully imaged the current distribution during delivery of microsecond high-voltage electroporation pulses. Their approach has the potential to enable monitoring of tumor coverage by the applied electric field during irreversible electroporation therapy and potentially other electroporation based therapies, such as electrochemotherapy and gene therapy by electroporation.

**Figure 1:**
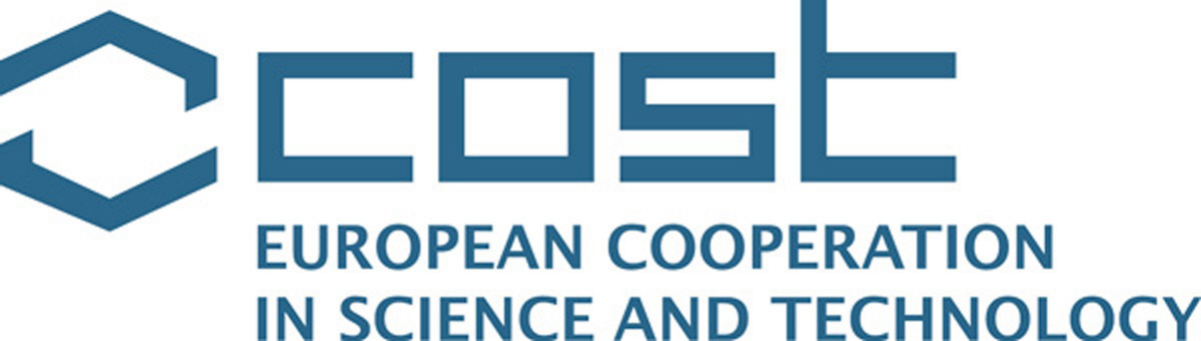
**COST logo**.

**Figure 2:**
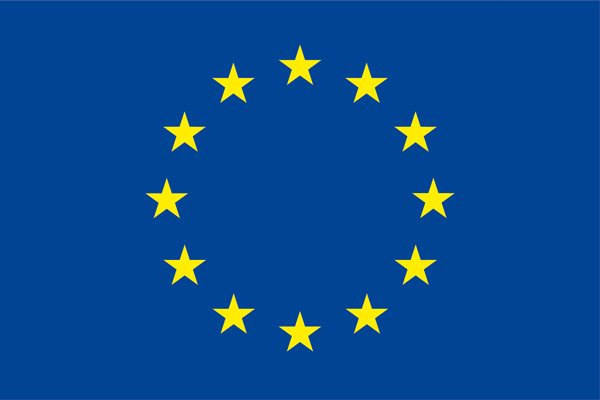
**EU logo (COST is supported by the EU Framework Programme Horizon 2020)**.

## Declaration

This publication was funded by COST (European Cooperation in Science and Technology). COST TD1104 Action EP4Bio2Med.

This article has been published as part of *BioMedical Engineering OnLine *Volume 14 Supplement 3, 2015: Select articles from the 6th European Conference of the International Federation for Medical and Biological Engineering (MBEC 2014). The full contents of the supplement are available online at http://www.biomedical-engineering-online.com/supplements/14/S3.
